# Serum IL-6 and IL-18 responses in COVID-19 in children: the role of vitamin D status in a cohort from Azerbaijan

**DOI:** 10.3389/fped.2026.1777675

**Published:** 2026-04-13

**Authors:** Ilhama Yelmar Huseynova, Alakbar Qazanfar Hasanov, Naila Hasan Sultanova, Fakhriyya Makhmud Mammadova, Tarana Qadir Taghi-zada, Aysel Azar Suleymanli, Ismayil Adil Gafarov

**Affiliations:** 1 II Department of Children's Diseases, Azerbaijan Medical University, Baku, Azerbaijan; 2 Department of Medical and Biological Physics, Azerbaijan Medical University, Baku, Azerbaijan

**Keywords:** children, COVID-19, IL-18, IL-6, vitamin D

## Abstract

**Introduction:**

Coronavirus disease 2019 (COVID-19) may trigger inflammatory responses in children, and vitamin D is known to play an important role in immune regulation.

**Methods:**

This study investigated the effects of COVID-19 infection and vitamin D status on inflammatory cytokine levels in children during the pandemic. A cohort of 170 children aged 1–17 years with PCR-confirmed SARS-CoV-2 infection was enrolled. Serum levels of IL-6 and IL-18 were analyzed in relation to vitamin D status and COVID-19 diagnosis.

**Results:**

Children with PCR-confirmed COVID-19 exhibited higher serum levels of IL-6 and IL-18 compared with healthy controls. Children with vitamin D deficiency had higher cytokine levels compared with those with normal vitamin D levels, although these differences were not statistically significant.

**Discussion:**

The findings suggest that low vitamin D levels may influence the increase in the studied cytokines; however, the current results do not fully confirm this relationship. These observations highlight the need for further large-scale and prospective studies to better understand the immunomodulatory role of vitamin D in pediatric COVID-19 patients.

## 1 Introduction

Coronavirus disease 2019 (COVID-19), caused by severe acute respiratory syndrome coronavirus 2 (SARS-CoV-2), emerged in Wuhan, China, in late 2019 and rapidly evolved into a global pandemic (1–3). The clinical spectrum of the disease ranges from mild respiratory symptoms to severe pneumonia and acute respiratory distress syndrome (ARDS). A notable feature of the pandemic has been the generally milder or asymptomatic presentation observed in pediatric patients compared to adults, a phenomenon potentially attributable to differences in immune system maturation.

A critical pathogenic mechanism underpinning severe COVID-19 is the “cytokine storm,” characterized by an excessive and dysregulated release of pro-inflammatory cytokines, including interleukin-6 (IL-6) and interleukin-18 (IL-18). This cascade can lead to uncontrolled systemic inflammation and multi-organ failure. As key mediators of immune regulation, cytokines are acutely elevated in response to viral replication in the upper respiratory mucosa. Despite growing evidence, the precise mechanisms governing cytokine dynamics in children remain incompletely elucidated.

Vitamin D has garnered significant interest for its immunomodulatory properties, influencing both innate and adaptive immune responses. Its active form, 1,25-dihydroxyvitamin D, enhances alveolar surfactant production, attenuates the expression of pro-inflammatory cytokines, and modulates the renin-angiotensin system in lung tissue (4–8). The interplay between COVID-19, vitamin D status, and the ensuing inflammatory response in the pediatric population warrants further exploration. This study represents the first investigation of this relationship in children from Azerbaijan. The primary objective was to evaluate the effects of SARS-CoV-2 infection and vitamin D deficiency on serum levels of IL-6 and IL-18 in children with PCR-confirmed COVID-19.

## 2 Patients and methods

### 2.1 Study design and participants

This retrospective study was conducted at the Children's Infectious Diseases Hospital in Baku, Azerbaijan, during 2022–2023, a period coinciding with ongoing pandemic lockdown measures. The study included hospitalized patients aged 1 month to 18 years diagnosed with COVID-19. All cases of SARS-CoV-2 infection were confirmed by polymerase chain reaction (PCR) testing of nasopharyngeal swabs, in accordance with WHO criteria. The standard clinical assessment for all patients included a detailed anamnesis, epidemiological history, physical examination, comprehensive laboratory testing, cytokine profiling, and radiological evaluation of the lungs.

The study protocol was reviewed and approved by the local Institutional Review Board, and informed consent was obtained from the parents or legal guardians of all participants.

**Exclusion criteria**: Children with rickets, bronchial asthma, autoimmune diseases, cystic fibrosis, primary or acquired immunodeficiency, other chronic diseases, mild or asymptomatic COVID-19, and multisystem inflammatory syndrome in children (MIS-C) were excluded from the study.

### 2.2 Laboratory analysis

Serum cytokine and vitamin D levels were measured using commercially available kits and a widely recognized analytical platform. Specifically, IL-6 and IL-18 concentrations were quantified using the Human IL-6 and Human IL-18 ELISA Kits (Invitrogen, USA), respectively. Vitamin D levels in all participants’ serum were measured using the enzyme-linked immunosorbent assay (ELISA) method utilizing the Stat Fax 4700 analyzer and reagent kits (Germany). The examinations were conducted during the acute phase of the disease, and serum IL-6 and IL-18 levels were measured within the first 24–48 h of hospitalization. Vitamin D status was classified based on serum 25(OH)D concentrations as follows: normal (30–100 ng/mL), insufficiency (20–29 ng/mL), deficiency (10–20 ng/mL), and severe deficiency (<10 ng/mL) (9).

### 2.3 Statistical analysis

Statistical analyses were performed using IBM SPSS Statistics, Version 26. Given the non-normal distribution of some variables, non-parametric tests were employed, including the Mann–Whitney U test and the Kruskal–Wallis H test. Correlations were assessed using Pearson's correlation coefficient. To evaluate the effects of multiple factors simultaneously, univariate analysis of variance (uANOVA) was conducted. A *p*-value of less than 0.05 was considered statistically significant. A *post-hoc* power analysis was performed, confirming sufficient statistical power for the main model [F(1,35) = 7.54, *p* = 0.009, partial *η*^2^ = 0.177, Cohen's f = 0.464].

## 3 Results

### 3.1 Demographic and clinical characteristics

A total of 170 children (92 boys and 78 girls) hospitalized with a diagnosis of COVID-19 were included in the study. All participants had PCR-confirmed SARS-CoV-2 infection and were clinically classified as having either moderate (121 patients; 71.2%) or severe disease (49 patients; 28.8%). Respiratory rate was assessed during hospitalization, and oxygen saturation (SpO₂) was measured using a Pulse Oximeter CMS50C device. Chest radiography revealed infiltrative pulmonary shadows of varying sizes. All examined patients demonstrated moderate to severe disease severity. The clinical presentation observed in this cohort was consistent with the typical manifestations of COVID-19. The most prominent symptoms among PCR-confirmed patients were fever and cough.

The majority of patients were urban residents (143 patients; 81.1%). The most frequently reported clinical symptoms included fever (155 patients; 91.2%), cough (123 patients; 72.4%), and muscle hypotonia (78 patients; 46.7%). Other reported symptoms included headache (27 patients; 15.9%), muscle pain (23 patients; 13.6%), cyanosis (22 patients; 13.2%), and loss of smell or taste (13 patients; 7.6%). Serum levels of IL-6 and IL-18 were analyzed in a subset of 75 patients. These patients were subsequently stratified according to vitamin D status and COVID-19 diagnosis for comparative analysis, as presented in Table 1.

**Table 1:** Grouping of children based on serum vitamin D levels and COVID-19 diagnosis.

Group	Category	*N*
I	Vitamin D normal	16
	Vitamin D ınsufficiency	47
	Vitamin D deficiency	12
II	Control	15
	COVID-19	75

### 3.2 Cytokine levels by vitamin D status

The levels of IL-6 and IL-18 according to vitamin D status are presented in Table 2. A trend was observed whereby children with vitamin D deficiency exhibited higher mean levels of IL-6 (3.925 pg/mL) and IL-18 (461.917 pg/mL) compared with children with normal vitamin D levels (IL-6: 3.242 pg/mL; IL-18: 349.178 pg/mL) or vitamin D insufficiency (IL-6: 2.694 pg/mL; IL-18: 352.146 pg/mL). However, these differences between vitamin D status groups did not reach statistical significance.

**Table 2:** Serum IL-6 and IL-18 levels in children with COVID-19 by vitamin D status.

Vitamin D status	Cytokine	Mean (pg/mL)	Std. error	95% CI
Normal	IL-6	3.242	0.539	2.170–4.315
	IL-18	349.178	30.391	288.752–409.603
Insufficiency	IL-6	2.694	1.489	0.000–5.654
	IL-18	352.146	83.923	185.285–519.007
Deficiency	IL-6	3.925	0.851	2.233–5.617
	IL-18	461.917	47.945	366.588–557.245

### 3.3 Cytokine levels by COVID-19 status

As shown in Table 3, a clear and statistically significant difference in cytokine levels was observed according to COVID-19 status. Children with PCR-confirmed COVID-19 demonstrated markedly higher mean levels of IL-6 (4.156 pg/mL) and IL-18 (442.624 pg/mL) compared with the healthy control group (IL-6: 1.664 pg/mL; IL-18: 268.346 pg/mL).

**Table 3:** Serum levels of IL-6 and IL-18 according to COVID-19 status in children.

COVID-19 status	Cytokine	Mean (pg/mL)	Std. error	95% CI
Control	IL-6	1.664	1.525	0.000–4.697
	IL-18	268.346	85.959	97.438–439.255
COVID-19	IL-6	4.156	0.402	3.358–4.955
	IL-18	442.624	22.632	397.626–487.621

### 3.4 ANOVA analysis

The results of the univariate analysis of variance (uANOVA) are presented in Table 4. The analysis evaluated the effects of Group I (vitamin D status), Group II (COVID-19 status), and their interaction on cytokine levels. The corrected model for IL-18 was statistically significant (*p* = 0.001). The intercept was significant for both cytokines, indicating overall model adequacy. The main effect of COVID-19 status demonstrated a non-significant trend for both IL-6 (*p* = 0.103) and IL-18 (*p* = 0.069). Neither the main effect of vitamin D status nor the interaction between the two factors reached statistical significance. A *post-hoc* power analysis was conducted based on the ANOVA results obtained for vitamin D levels between the two age groups. According to the SPSS output, the ANOVA test yielded F(1,35) = 7.54; *p* = 0.009, with partial *η*^2^ = 0.177 and Cohen's f = 0.464.

**Table 4:** u-ANOVA results for IL-6 and IL-18.

Source	Cytokine	Type III SS	df	Mean square	F	Sig. (p)
Corrected model	IL-6	84.875	4	21.219	2.443	0.053
	IL-18	552,058.696	4	138,014.674	5.003	0.001
Intercept	IL-6	141.522	1	141.522	16.294	<0.001
	IL-18	2,109,940.011	1	2,109,940.011	76.488	<0.001
Group I (COVID-19 status)	IL-6	23.542	1	23.542	2.710	0.103
	IL-18	93,847.306	2	46,923.653	1.701	0.190
Group II (Vit. D status)	IL-6	2.073	2	1.036	0.119	0.888
	IL-18	8,031.541	1	8,031.541	0.291	0.591
Group I × Group II	IL-6	1.332	1	1.332	0.153	0.696
	IL-18	22,796.123	1	22,796.123	0.826	0.366

Group I refers to COVID-19 status (Control, COVID-19). Group II refers to vitamin D status (Normal, Insufficient, Deficient).

These results indicate that the analysis had adequate statistical power to detect the observed effect size at the 5% significance level.

### 3.5 Comparison with international studies

A comparison of our findings with those reported in international pediatric studies is presented in Table 5. Our cohort from Azerbaijan demonstrated a high prevalence of vitamin D deficiency (66.7% when deficiency and insufficiency were combined). Notably, the mean IL-6 level observed in our study (4.16 pg/mL) was lower than values reported in studies from Italy, Egypt, and Turkey. These differences may reflect variations in disease severity, timing of sample collection, or population-specific characteristics.

**Table 5:** Comparison of IL-6 and IL-18 levels with international pediatric studies.

Study	Country	N	IL-6 (pg/mL)	IL-18 (pg/mL)	Vit D deficiency	*p*-value
Present study	Azerbaijan	75	4.16	442.6	66.7%	<0.001
Curatola 2021 (13)	Italy	62	23.7	NR	45%	<0.01
Shafiek 2021 (12)	Egypt	89	15.8	NR	58%	<0.001
Kamil 2020 (8)	Turkey	85	12.3	NR	71%	<0.001
Wang 2024* (5)	15 countries	2,143	8.2–31.4	Limited	12–64%	Variable

*NR, Not reported; meta-analysis.

**Figure 1:**
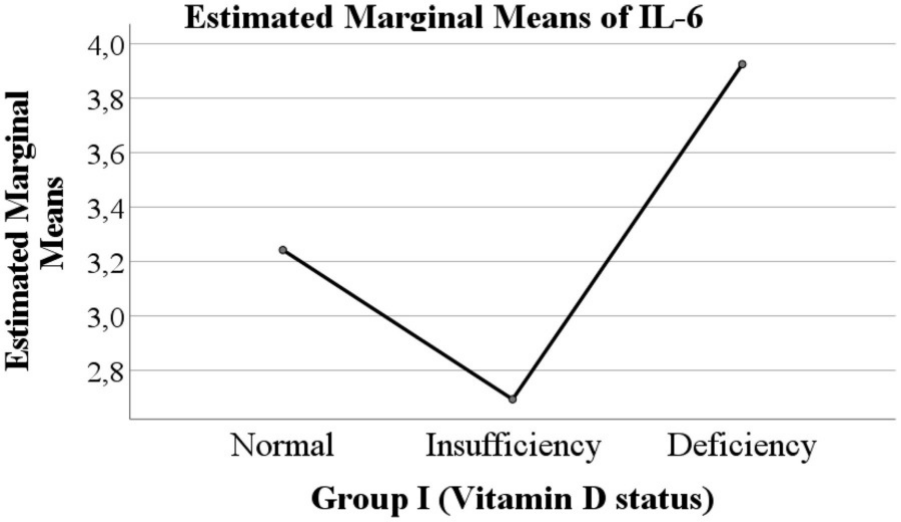
Serum IL-6 concentrations in children according to vitamin D status.

**Figure 2:**
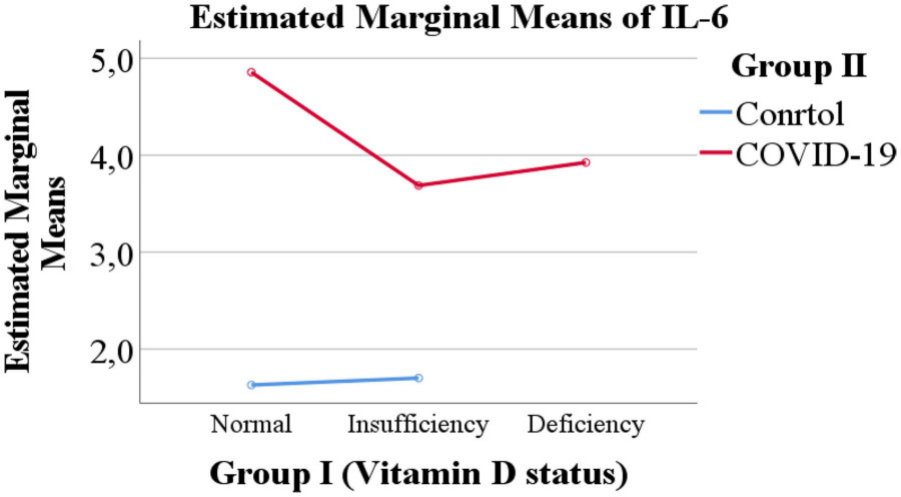
Serum IL-6 concentrations in children stratified by vitamin D status and COVID-19 infection.

**Figure 3:**
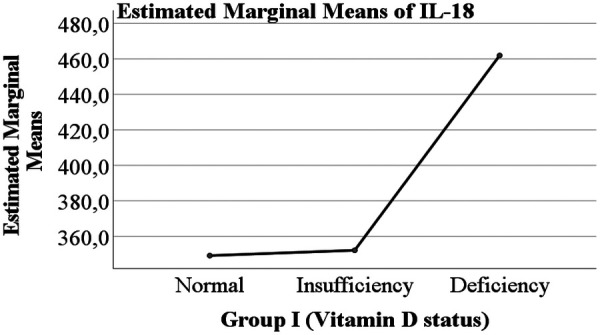
Serum IL-18 concentrations in children according to vitamin D levels.

**Figure 4:**
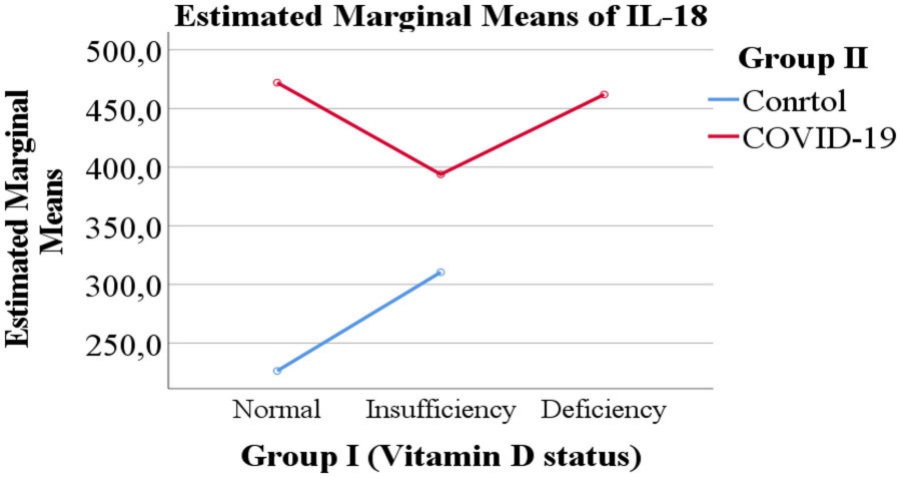
Serum IL-18 concentrations in children stratified by vitamin D status and COVID-19 infection.

Overall, both COVID-19 status and vitamin D levels were evaluated as potential factors influencing the levels of IL-6 and IL-18 (Figures 1–4).

## 4 Discussion

This study evaluated serum levels of the pro-inflammatory cytokines IL-6 and IL-18 in pediatric COVID-19 patients in relation to their vitamin D status. Our findings confirm that SARS-CoV-2 infection in children is associated with a significant elevation of these cytokines compared to healthy controls, aligning with the established pathophysiology of COVID-19 involving immune dysregulation and the cytokine storm phenomenon (10–16).

The central focus of our investigation was the potential modulatory role of vitamin D. We observed a consistent trend wherein children with vitamin D deficiency exhibited higher mean levels of both IL-6 and IL-18 compared to their counterparts with sufficient levels. This trend is biologically plausible, given vitamin D's known role in suppressing pro-inflammatory pathways and promoting immune homeostasis. However, this association did not achieve statistical significance in our cohort. This lack of significance may be attributable to several factors, including the relatively small sample size in the vitamin D deficient subgroup (*n* = 12), which limits statistical power. Furthermore, the exclusive inclusion of children with moderate to severe disease may have reduced the variability needed to detect a moderating effect of vitamin D, as the inflammatory response in these patients is already pronounced.

When contextualized within the international literature (Table 5), our cohort exhibited a high prevalence of vitamin D deficiency, yet mean IL-6 levels were notably lower than those reported in other studies. This discrepancy could be influenced by ethnic and genetic factors, differences in predominant SARS-CoV-2 variants, or variations in clinical management. The cytokine storm, driven by IL-6 and IL-18, can cause significant damage to the alveolar epithelium, impair gas exchange, and contribute to the development of ARDS. The trend we observed suggests that vitamin D deficiency might exacerbate this inflammatory cascade, even if the effect is modest. Therefore, screening for vitamin D status in pediatric COVID-19 patients could still be valuable for identifying children who might be at a marginally increased risk of a heightened inflammatory state (17–21).

## 5 Conclusion

In summary, this study provides clear evidence that COVID-19 in children is associated with significantly elevated levels of the pro-inflammatory cytokines IL-6 and IL-18. While vitamin D deficiency was associated with a non-significant trend toward further increases in these cytokines, the potential for vitamin D to modulate the inflammatory response in pediatric COVID-19 should not be dismissed. The trends observed here highlight the need for more extensive, prospective studies to clarify this relationship. Future research, particularly interventional trials assessing the impact of vitamin D supplementation on cytokine profiles and clinical outcomes in pediatric COVID-19, is essential to determine whether a causal and therapeutically relevant relationship exists.

## 6 Study limitations

This study has several limitations that should be considered when interpreting the results. Firstly, patient recruitment was challenged by strict quarantine measures and parental hesitancy, constraining the overall sample size. Secondly, the study was restricted to children with moderate and severe COVID-19; the inclusion of mild or asymptomatic cases could have provided a more comprehensive understanding of the cytokine response across the disease spectrum. Thirdly, the sample size, particularly for the subgroup with vitamin D deficiency, was limited, reducing the statistical power to detect potentially small but clinically meaningful effects. Due to the limited sample size, a full multivariable model could have reduced statistical power. Nevertheless, subgroup analyses by age, sex, nutritional status, and comorbidities were conducted, and the results did not show statistically significant changes. Finally, widespread lockdowns and associated lifestyle changes during the pandemic may have influenced vitamin D levels in the general population, potentially introducing a confounding variable that was not fully accounted for.

## Data Availability

The data that support the findings of this study are not publicly available due to privacy and ethical restrictions concerning patient information. The data is available from the corresponding author, Ilhama Yelmar Huseynova, upon reasonable request.
